# Synthesis of embryonic tendon-like tissue by human marrow stromal/mesenchymal stem cells requires a three-dimensional environment and transforming growth factor β3

**DOI:** 10.1016/j.matbio.2010.08.005

**Published:** 2010-10

**Authors:** Zoher Kapacee, Ching-Yan Chloé Yeung, Yinhui Lu, David Crabtree, David F. Holmes, Karl E. Kadler

**Affiliations:** Wellcome Trust Centre for Cell-Matrix Research, University of Manchester, Faculty of Life Sciences, Michael Smith Building, Oxford Road, Manchester M13 9PT, England, United Kingdom

**Keywords:** BM-MNC, Bone marrow-derived mononuclear cell, MSC, Mesenchymal/marrow stromal stem cell, Collagen, Fibrin, Differentiation, Signaling, Forces

## Abstract

Tendon-like tissue generated from stem cells *in vitro* has the potential to replace tendons and ligaments lost through injury and disease. However, thus far, no information has been available on the mechanism of tendon formation *in vitro* and how to accelerate the process. We show here that human mesenchymal stem cells (MSCs) and bone marrow-derived mononuclear cells (BM-MNCs) can generate tendon-like tissue in 7 days mediated by transforming growth factor (TGF) β3. MSCs cultured in fixed-length fibrin gels spontaneously synthesized narrow-diameter collagen fibrils and exhibited fibripositors (actin-rich, collagen fibril-containing plasma membrane protrusions) identical to those that occur in embryonic tendon. In contrast, BM-MNCs did not synthesize tendon-like tissue under these conditions. We performed real-time PCR analysis of MSCs and BM-MNCs. MSCs upregulated genes encoding type I collagen, TGFβ3, and Smad2 at the time of maximum contraction of the tendon-like tissue (7 days). Western blot analysis showed phosphorylation of Smad2 at maximum contraction. The TGFβ inhibitor SB-431542, blocked the phosphorylation of Smad2 and stopped the formation of tendon-like tissue. Quantitative PCR showed that BM-MNCs expressed very low levels of TGFβ3 compared to MSCs. Therefore we added exogenous TGFβ3 protein to BM-MNCs in fibrin gels, which resulted in phosphorylation of Smad2, synthesis of collagen fibrils, the appearance of fibripositors at the plasma membrane, and the formation of tendon-like tissue. In conclusion, MSCs that self-generate TGFβ signaling or the addition of TGFβ3 protein to BM-MNCs in fixed-length fibrin gels spontaneously make embryonic tendon-like tissue *in vitro* within 7 days.

## 1 Introduction

Adult mesenchymal stem cells (MSCs or marrow stromal cells) are a unique population of cells that were first discovered as distinct colony-forming cells that adhere to cell culture plastic (Friedenstein et al., 1970). They are characterized by the ability to self-renew and differentiate into adult cell types including osteoblasts, chondrocytes, adipocytes and fibroblasts (see (Caplan, 2007) and references therein). Multipotency of MSCs has been demonstrated *in vitro* by chemical induction (e.g. by adding exogenous growth factors) or by adherence to surfaces with differing elasticity (Engler et al., 2006). In an early study by Hinz and co-workers, stiff silicone substrates were shown to assist the *trans*-differentiation of fibroblasts to myofibroblasts (Hinz et al., 2001). The study by Engler and co-workers showed that human MSCs cultured on a stiff surface adopted bone and muscle phenotypes whereas cells adhering to a soft surface readily differentiated down a neurogenic lineage. Interestingly, a tendon phenotype was not reported, which implies that the differentiation of MSCs to a tendon lineage has additional culture requirements that are not exclusively dependent on the stiffness of the culture surface. The ability to make tendons *in vitro* from bone marrow-derived cells or stem cells would be expected to have a major impact on the treatment of musculoskeletal injuries. As summarized by Butler et al. (2008), more than 32 million traumatic and repetitive motion injuries to tendons and ligaments occur annually in the USA (Schoen, 2005) with rotator cuff and iatrogenic tendon injuries of the anterior cruciate ligament being among the most common. Tendons are rich in extracellular matrix (ECM) and have relatively few cells, which helps to explain why tendons heal slowly and why re-establishment of normal function after surgery remains challenging. Furthermore, adhesion between the surface of an injured tendon and the surrounding sheath is an unwanted, but often unavoidable, complication (Wong et al., 2009). Therefore, new strategies are needed to encourage regeneration of injured tendons and to replace tendons (and ligaments) with tendon-like tissues grown rapidly in the laboratory.

Chen and co-workers showed that embryonic stem cells (ESCs) can be used to produce engineered embryonic tendons (Chen et al., 2009). The engineered tendons were produced by culturing the cells in 2D sheets, rolling the sheets into a cylinder, and mechanically loading the cylinders for 2 weeks. Other studies have shown that MSCs seeded in collagen gels under static or dynamic tension are a model for studying the potential of MSCs in regenerating a tendon- or ligament-like tissue (Kuo et al., 2008). These studies raise the interesting possibility that the shape of the cell, or the shape of the culture environment, is important in defining the tendon phenotype. However, the precise mechanisms involved in MSC-to-tendon transition remain poorly understood.

Transforming growth factor (TGF) β signaling is a major regulator of the differentiation and growth of connective tissues. TGFβs are a subfamily of bioactive polypeptides within the TGFβ superfamily of growth factors that include growth differentiation factors (GDFs), bone morphogenetic proteins (BMPs), nodal, activins, and inhibins. Three TGFβs (TGFβ1–3) occur in mammals and birds. TGFβs are synthesized as a small latent complex (SLC) that comprises the mature dimeric TGFβ non-covalently associated with its own latency-associated peptide (LAP). Although the LAP is cleaved by furin-like proteases in the secretory pathway it remains non-covalently bound to TGFβ in the SLC. The SLC can be secreted as part of a large latent complex (LLC) in which the LAP is disulphide bound to a latent TGFβ binding protein (Rifkin, 2005; Saharinen and Keski-Oja, 2000) (LTBP). The LTBP (with bound, inactive TGFβ) can be sequestered in the extracellular matrix (ECM) by transglutaminase crosslinking (Nunes et al., 1997). The active TGFβ can be released by proteolytic (e.g. BMP1 (Ge and Greenspan, 2006)) or non-proteolytic (e.g. involving integrins αvβ6 (Annes et al., 2004; Munger et al., 1999), reactive oxygen species (Amarnath et al., 2007), cell contraction (Wipff et al., 2007), extremes of pH (Annes et al., 2003), or thrombospondin-1 (Crawford et al., 1998)) mechanisms. Other studies have demonstrated activation of TGFβ1 by a hybrid of proteolytic and non-proteolytic activation in which SLC bound to αvβ8 results in the membrane type 1 (MT1)-MMP-dependent release of active TGFβ1, leading to autocrine and paracrine effects on cell growth and matrix production (Mu et al., 2002). The bioavailability of TGFβs can be further regulated in the ECM through reversible interactions with proteins including decorin and biglycan, which have a much weaker interaction with TGFβ3 than with TGFβ1 (Baker et al., 2009). The biological effects of TGFβs are exerted by signal transduction through transmembrane serine/threonine kinase receptors (TGFBR1 (type I) and TGFBR2 (type II)) leading to Smad-dependent and Smad-independent downstream processes (see (Attisano and Wrana, 2002; Chang et al., 2002; de Caestecker, 2004; Massague et al., 2005; Xu and Attisano, 2000) and references therein). Pryce and co-workers showed that TGFβ signaling induces the tendon progenitor marker, scleraxis (Schweitzer et al., 2001), and that disruption of TGFβ signaling in Tgfb2−/−;Tgfb3−/− double mutant mouse embryos or through the inactivation of TGFBR2 results in loss of most tendons and ligaments in the limbs, trunk, tail and head (Pryce et al., 2007). Therefore, TGFβ signaling is essential for the maintenance of tendon progenitor cells.

In our study present here, we wanted to know if MSCs and bone marrow-derived mononuclear cells (BM-MNCs) could be induced to make a tendon-like tissue *in vitro* and if we could manipulate TGFβ signaling to enhance the process. We had shown previously that embryonic tendon cells cultured in fixed-length three-dimensional (3D) tensioned fibrin gels maintained their embryonic tendon-like morphology*.* The cells assembled parallel bundles of narrow-diameter collagen fibrils and exhibited fibripositors (Kapacee et al., 2008), which are plasma membrane protrusions that contain narrow-diameter collagen fibrils (Canty et al., 2004) and which are abundant in embryonic tendon. Fibripositors can be found in flexor tendon adhesions (Wong et al., 2009) and in embryonic cornea, ligament, periosteal bone, and skin (Lu and Kadler, unpublished results). However, they are most abundant and readily apparent by electron microscopy of embryonic tendon (Canty et al., 2004). Fibrin gels have been used to culture a variety of cells, including skeletal muscle (Huang et al., 2006, 2005), cardiac muscle (Huang et al., 2007), and collagen-producing smooth muscle cells (Hecker et al., 2005). It occurred to us that 3D fixed-length tensioned fibrin gels could be used to evaluate the differentiation potential of MSCs and BM-MNCs to a tendon lineage.

## 2 Results

### 2.1 Characterization of MSCs

The two strains of MSCs from two different people were characterized using flow cytometry and lineage induction.

#### 2.1.1 Flow cytometry

Strains L1 and L2 were assessed for expression of selected surface markers. All data were collected from live cells by selective gating (an example is shown in Supplemental 1). Analysis showed that the cells expressed CD44, CD90 and CD105, which are cell surface markers expressed by MSCs (for review of stem cell markers see Phinney and Prockop (2007)). Furthermore, the cells lacked CD34 as well as the monocyte surface marker CD11b (Colter et al., 2001).

#### 2.1.2 Chemical induction

Strains L1 and L2 were assessed for the ability to undergo adipogenic, osteogenic and chondrogenic differentiation (see Supplemental 2). Within 21 days of adipogenic induction MSCs examined by cytohistochemistry using Oil Red O displayed lipid droplets and by immunofluorescence expressed fatty acid binding protein (FABP)-4, a cytosolic chaperone present in adipocytes (Supplemental 2 A–D). Within 21 days of osteogenic induction MSCs examined by alizarin red S staining showed evidence of calcified deposits and by immunofluorescence expressed elevated levels of osteocalcin (Supplemental 2 E–H). Within 28 days of chondrogenic induction pellets of MSCs were larger and stained positively for sulphated proteoglycans as displayed by safranin O (Supplemental 2 I–L).

### 2.2 MSCs in uniaxial tensioned 3D culture resemble embryonic tendon cells

**Fig. 1:**
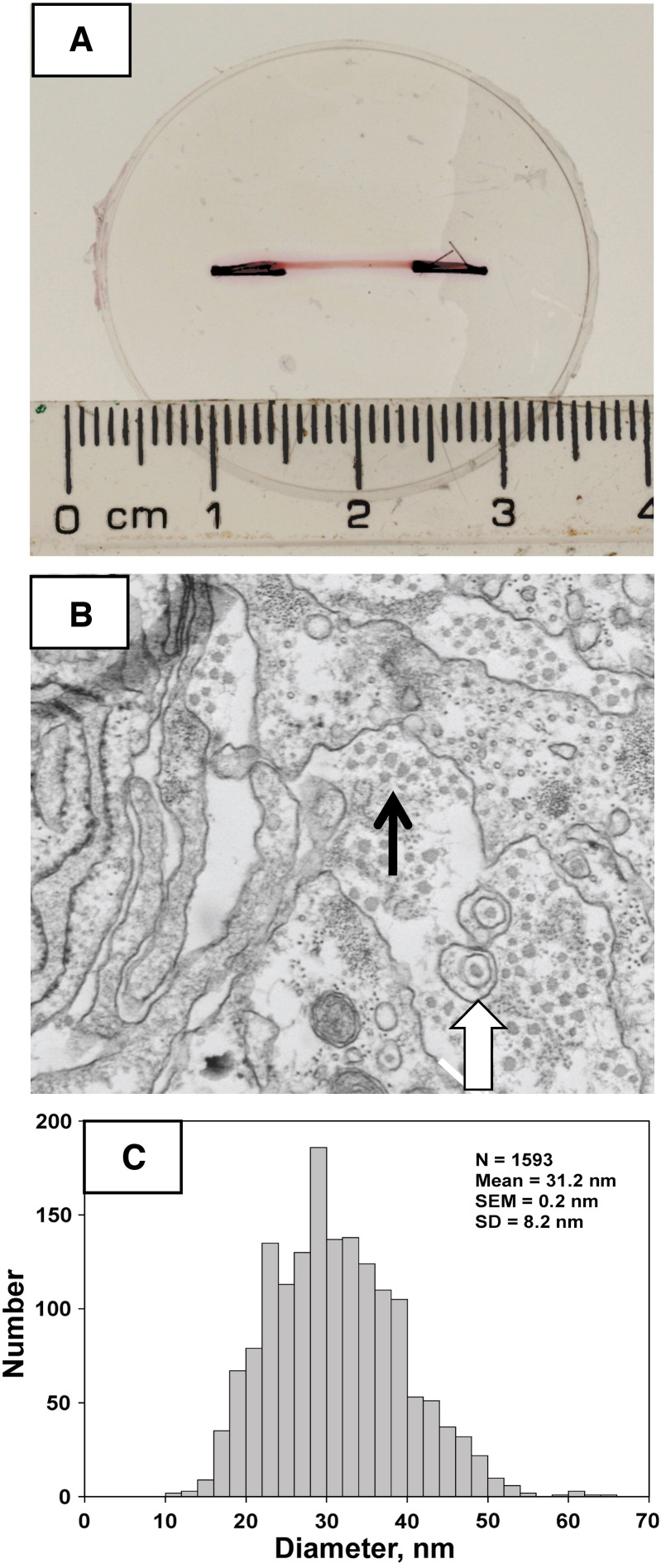
Ultrastructural analysis of tissue constructs assembled by MSCs. A, Plan view of a fully-contracted construct (pink) between fixed position posts containing sutures (black). The construct is pink because of the phenyl red in the culture medium. B, Transmission electron microscopy shows the presence of close-packed narrow-diameter (~ 30 nm) collagen fibrils (black arrow) and fibripositors (white block arrow). C, The distribution of diameters of the collagen fibrils in the tendon-like constructs.

Fibrin gels were seeded with MSCs using methods previously used to culture chick embryonic tendon cells (Kapacee et al., 2008). During 7 days in culture, the cells contracted the gels to form a construct between the fixed-positioned minutien pins (Fig. 1A). Transmission electron microscopy showed that the constructs were populated with cells that exhibited fibripositors (Canty et al., 2004) and were embedded in a rich ECM comprising narrow-diameter collagen fibrils (Fig. 1B). The fibrils had a mean diameter of 31.2 nm ± 8.2 (S.D., N = 1593) (Fig. 1C), which was similar to the fibril diameters observed in E13 chick tendon (~ 36 nm) and E13 chick tendon cells cultured in the same 3D fibrin gels (~ 34 nm) (see (Kapacee et al., 2008)).

We considered the possibility that tendon induction had occurred because of exposure of the MSCs to thrombin during the first few minutes of forming the fibrin gel. Also, we wanted to know if fibrin *per se* was responsible for induction of narrow collagen fibrils and fibripositors at the plasma membrane. In one approach we exposed MSCs to thrombin (under identical conditions used in the formation of a tendon-like construct) and cultured the cells on pre-formed fibrin gels. As shown in Supplemental 3A, the cells adhered to the fibrin but did not synthesize collagen fibrils. Next, we cultured MSCs (not treated with thrombin) on pre-formed fibrin gels. As shown in Supplement 3B, the cells did not synthesize collagen fibrils. Electron microscopy of the cells and dye exclusion assays showed that the cells were viable in culture. The results showed that exposure to thrombin or culturing the cells ‘on’ fibrin was not responsible for tendon induction of MSCs. Therefore, culturing the cells within a 3D fibrin gel appeared to be an important prerequisite for rapid tendon induction.

### 2.3 MSCs cultured with uniaxial tension in tendon-like constructs lose the ability to respond to osteogenic induction

Preliminary data from flow cytometry showed that MSCs cultured in the tendon-like constructs lose expression of CD44. These data provided the first indications that exposure to tension in the tendon-like constructs changed the ‘stemness’ of the cells. Therefore, we released MSCs from tendon-like constructs and cultured the cells for a further 21 days in medium used for osteogenic induction. Staining with alizarin red S and immunofluorescence for osteocalcin showed the absence of calcified deposits. Therefore, the cells appear to have a dampened ability to undergo osteogenic induction (Supplemental 2M–P).

### 2.4 MSCs upregulate collagen gene expression in the 3D tendon-like constructs

**Fig. 2:**
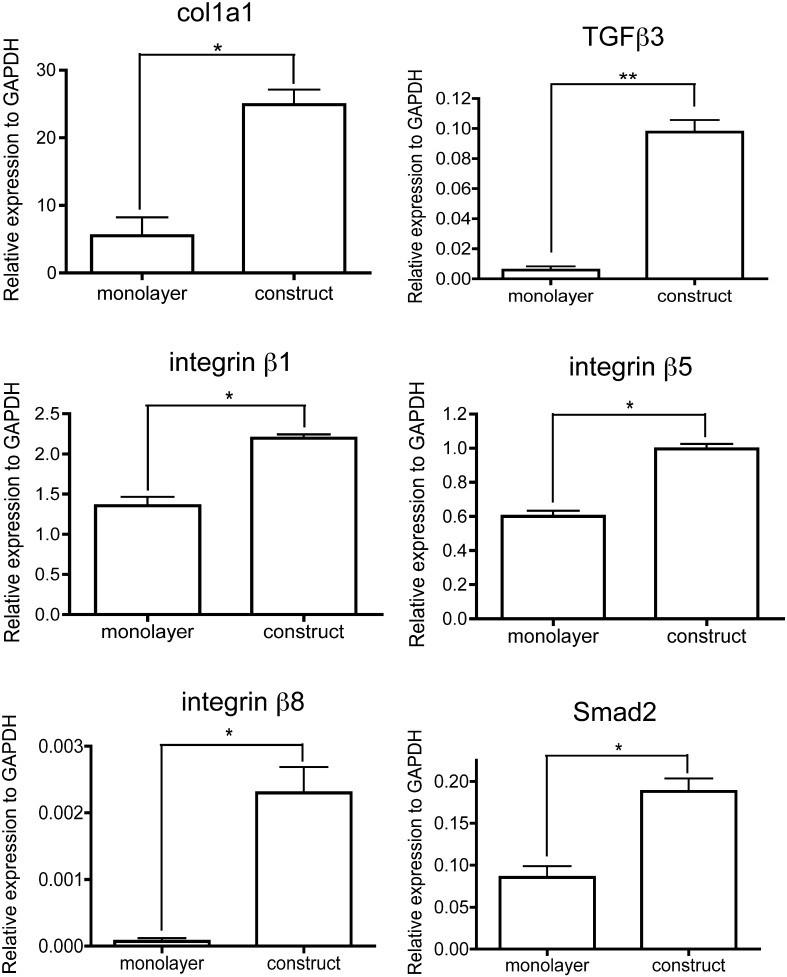
Quantitative PCR comparison of gene expression in MSCs cultured in monolayer and in tendon-like constructs, relative to expression of the gene for GAPDH. * denotes p value < 0.05. ** denotes p value < 0.005.

To obtain an insight into how MSCs assemble a tendon-like matrix and if TGFβ signaling was involved, we used quantitative PCR (Q-PCR) to compare the levels of expression of selected genes when the cells were cultured in monolayer and in 3D fibrin constructs. As shown in Fig. 2, the levels of gene expression for col1a1 (which encodes the α1(I) chain of type I collagen), TGFβ3, integrin β1, integrin β5, integrin β8 and Smad2 were significantly higher in the tendon-like constructs compared to cells cultured in monolayer. The levels of gene expression for TGFβ1, fibronectin, tenascin C, fibromodulin, decorin, biglycan, scleraxis (a transcription factor expressed in tendon (Asou et al., 2002; Schweitzer et al., 2001)), fibrillin, integrin αv, latent TGFβ binding protein (LTBP)-1, tenascin C, BMP-1 (which is involved in collagen fibril assembly and TGFβ activation) and thrombospondin-1 were not significantly different in cells cultured on tissue culture plastic and cells cultured in the constructs (Supplemental 4).

### 2.5 Evidence for Smad-dependent TGFβ signaling in the tendon-like constructs

**Fig. 3:**
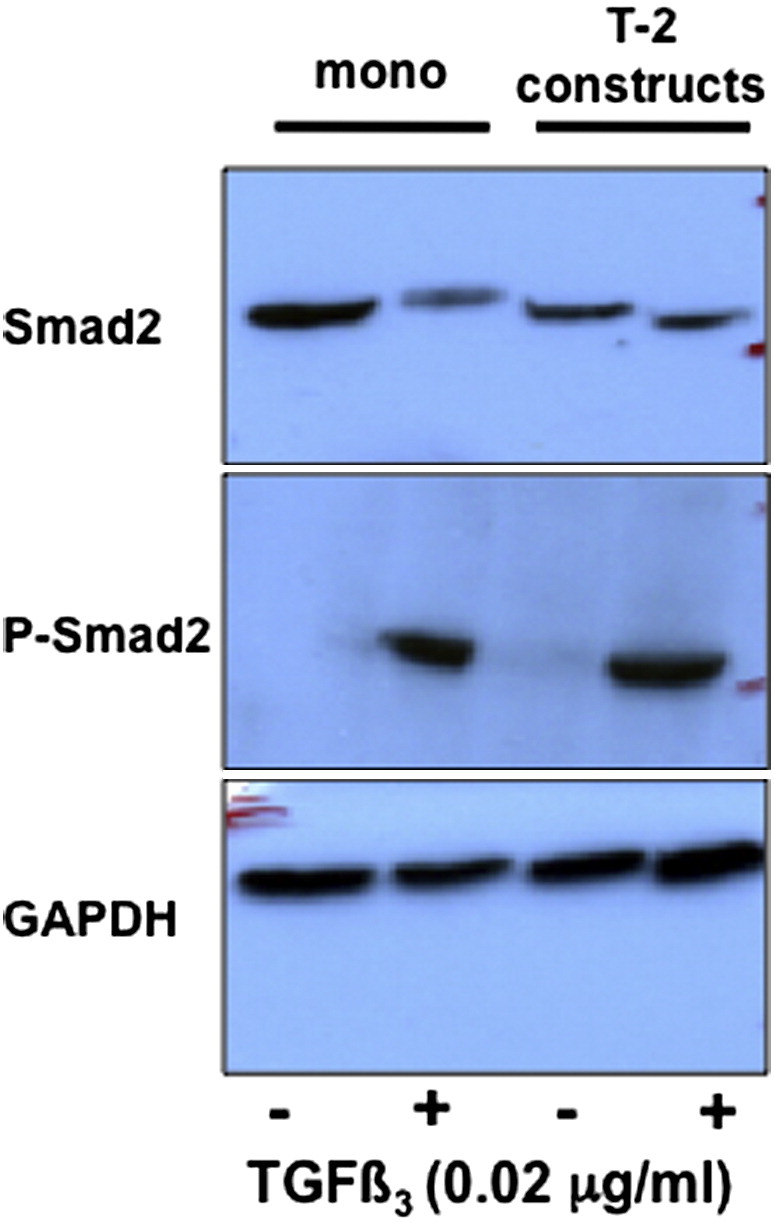
Analysis of Smad-dependent TGFβ signaling in MSCs. MSCs were grown on tissue culture plastic and on fibrin gels for 5 days (2 days before contraction, denoted by T-2) in the presence and absence of exogenous TGFβ3. Cell lysates were examined by Western blot analyses using antibodies to Smad2, P-Smad2, and glyceraldehyde phosphate dehydrogenase (GAPDH). GAPDH was used as a loading control.

The upregulation of TGFβ3 gene expression in cells in tendon-like constructs compared to monolayer prompted us to determine if Smad-dependent signaling occurs in the tendon constructs. We isolated proteins from MSCs in monolayer and from MSCs 2 days prior to full contraction i.e. T-2 or 5 days after cell seeding when the constructs are still gel-like and flimsy. Using Western blot analysis we showed that Smad2 was expressed in MSCs in monolayer and in T-2 fibrin gels but there was no evidence of phospho-Smad2 (P-Smad2) (Fig. 3), which is an indicator of Smad-dependent TGFβ signaling (Massague et al., 2005). To determine if the cells were competent for Smad-dependent TGFβ signaling, we added exogenous TGFβ3 and performed Western blot analysis for P-Smad2. As shown in Fig. 3, the cells responded to the addition of exogenous of TGFβ3 by phosphorylating Smad2.

**Fig. 4:**
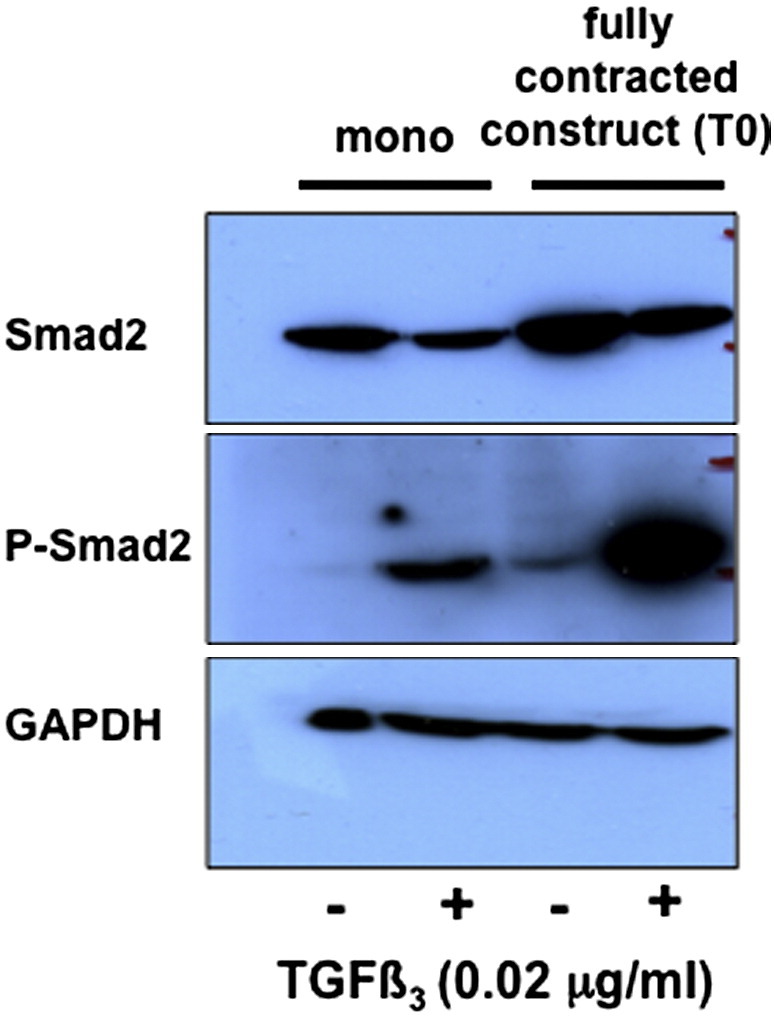
Analysis of Smad-dependent TGFβ signaling in stem cells in monolayer culture and in tendon-like constructs. MSCs were grown on tissue culture plastic and in constructs for 7 days in the presence and absence of exogenous TGFβ3. Cell lysates were examined by Western blot analyses using antibodies to Smad2, P-Smad2, and glyceraldehyde phosphate dehydrogenase (GAPDH). GAPDH was used as a loading control.

Next, we used Western blot analysis to determine the phosphorylation state of Smad2 in cells in fully-contracted constructs. As shown in Fig. 4, Smad2 is phosphorylated in cells in fully-contracted constructs in which the fibrin is replaced by collagen fibrils (see Fig. 2). P-Smad2 was present in the absence of exogenous TGFβ3, however, the addition of exogenous TGFβ3 increased the levels of P-Smad2 relative to GAPDH.

### 2.6 Inhibition of TGFβ signaling in MSCs stops the formation of tendon-like constructs

**Fig. 5:**
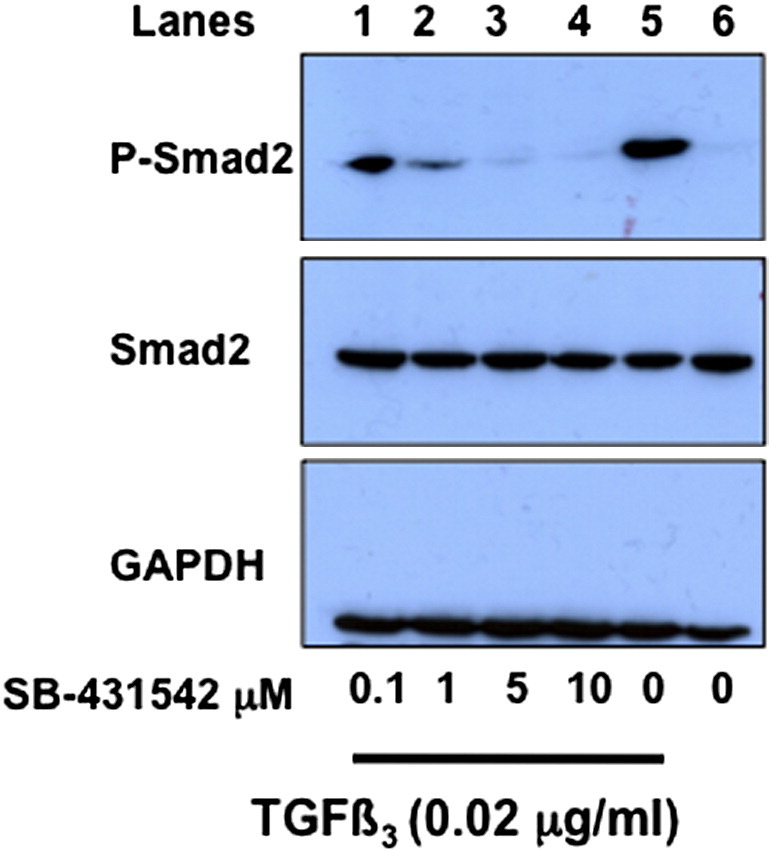
SB-431542 is effective at inhibiting Smad2-dependent signaling in MSCs. MSCs in monolayer were incubated with exogenous TGFβ3 and with the TGFβ inhibitor, SB-431542, at the concentrations shown. Cell lysates were examined by Western blot analyses using antibodies to Smad2, P-Smad2, and glyceraldehyde phosphate dehydrogenase (GAPDH). GAPDH was used as a loading control.

The fact that TGFβ3 levels were elevated 15-fold (p < 0.001) in constructs (Fig. 2) and because Smad2 is phosphorylated in fully-contracted constructs (Fig. 4), we wanted to know the effects of inhibiting TGFβ on the formation of the constructs. SB-431542 is a small molecule potent inhibitor of activin receptor-like kinase (the TGFβ type 1 receptor) (Inman et al., 2002) that is effective in inhibiting TGFβ signaling in a variety of cells, including MSCs (Droguett et al., 2010; Popova et al., 2010; Stewart et al., 2010). We added SB-431542 to the culture medium of MSCs in monolayer in the presence and absence of exogenous TGFβ3 (0.02 μg/ml) and used Western blot analysis to determine the presence of Smad2 and P-Smad2 in the cells. As shown in Fig. 5, SB-431542 was highly effective in inhibiting the phosphorylation of Smad2. Next, we added SB-431542 (10 μM) to the culture medium at the time of setting up the tendon-like constructs. The results showed that in all of 12 attempts (2 × 6 well plates), none formed constructs and fibrin was digested, leaving cells in monolayer.

### 2.7 BM-MNCs could be induced to make a tendon-like construct only in the presence of exogenous TGFβ3

**Fig. 6:**
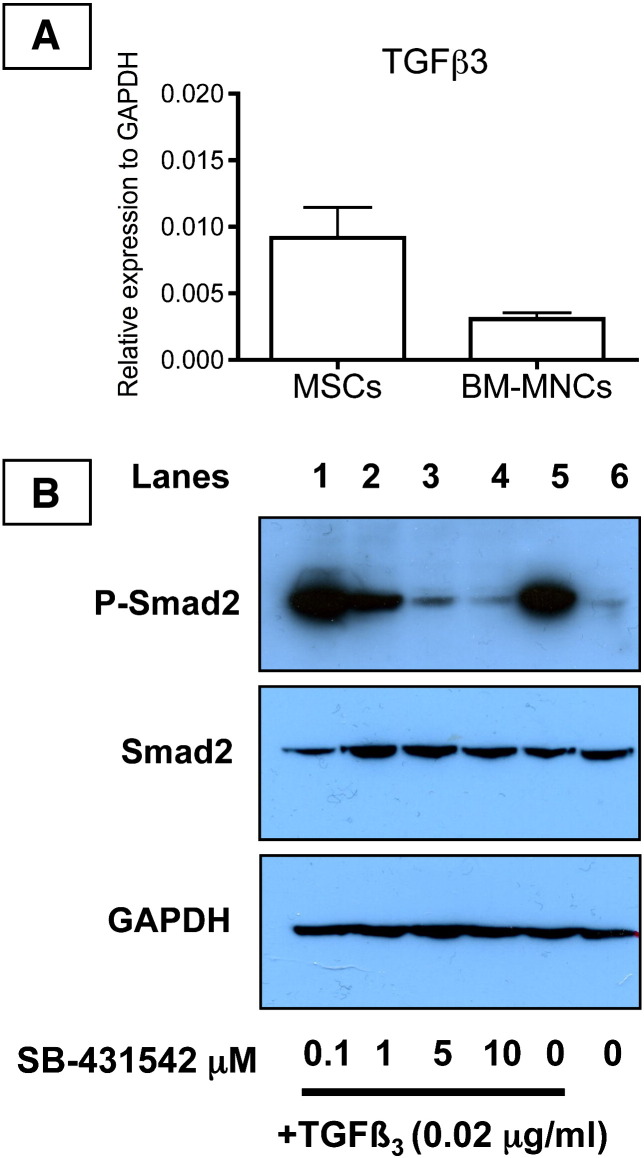
BM-MNCs express low levels of TGFβ3 compared to MSCs but are responsive to exogenous TGFβ3. A, Q-PCR comparison of TGFβ3 mRNA levels in MSCs and BM-MNCs. B, BM-MNCs grown for 14 days in fibrin constructs and the cell lysates examined by Western blot analysis for the presence of Smad2 and phospho-Smad2 (P-Smad2), using glyceraldehyde phosphate dehydrogenase (GAPDH) was used as a loading control. Lanes 1–5, TGFβ3 (0.02 μg/ml) was added to the constructs in the presence of SB-431542 at the concentrations shown.

**Fig. 7:**
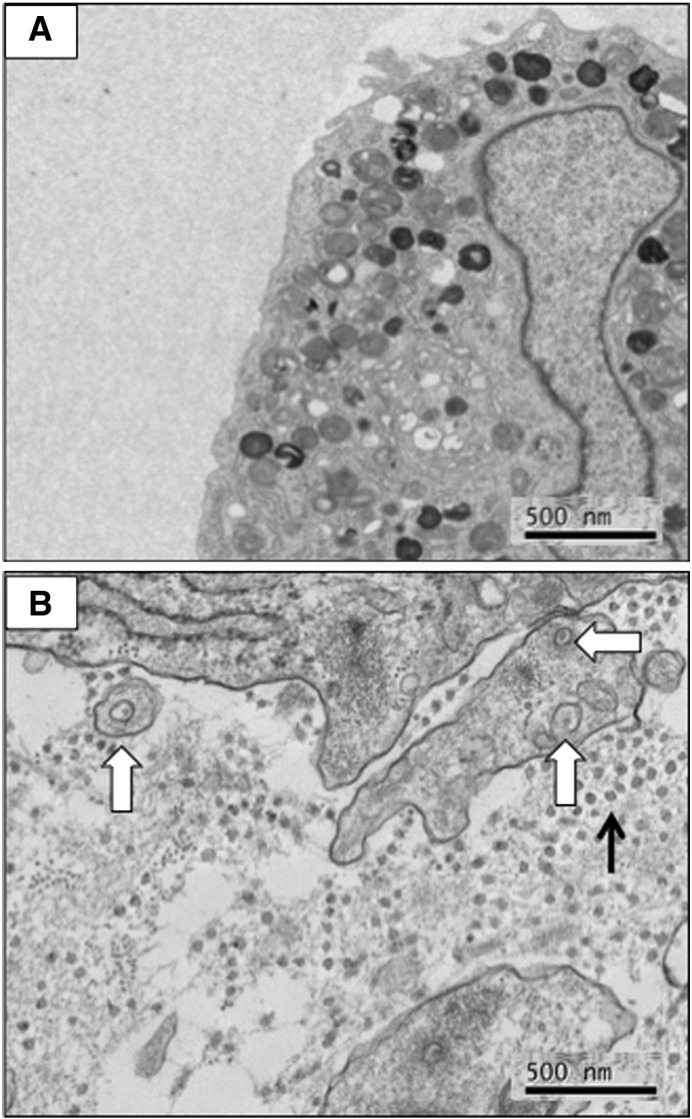
Exogenous TGFβ3 is required to generate a tendon-like construct from BM-MNCs. A, Electron microscopy of a BM-MNC construct. The construct contains cells within a fibrin matrix. The cells exhibited numerous vacuoles. B, Electron microscopy of a BM-MNC construct incubated with TGFβ3 (0.02 μg/ml). The cells have generated a matrix containing narrow-diameter collagen fibrils (black arrow), some of which are contained with fibripositors (white block arrows).

TGFβ1 and TGFβ3 bind to the same TGFβ type 1 receptor, therefore, it was not possible from the SB-431542 experiments to determine if the inhibition of construct formation was specific to TGFβ3. Also, the high conservation in amino acid sequence between TGFβ isoforms deterred us from attempting to quantitate the levels of TGFβ3 or compare the levels to TGFβ1, TGFβ2 and TGFβ3. Still further, detection of TGFβ isoforms by Western blotting would not have provided useful information on the bioavailability or bioactivity of the molecules. However, in our analyses of different cell strains we noted that BM-MNCs expressed very low levels of TGFβ3 mRNA as determined by Q-PCR (Fig. 6A). Western blot analysis showed that the cells were responsive to exogenous TGFβ3 as shown by the phosphorylation of Smad2 in the presence of increasing concentrations of TGFβ3 (Fig. 5). Furthermore, the phosphorylation of Smad2 was inhibited by SB-431542. Therefore, the BM-MNCs responded similarly to exogenous TGFβ3 and SB-431542 as did MSCs. However, in contrast to MSCs, BM-MNCs were slow to form a tendon-like construct (10 days) and the construct was mostly comprised of undigested fibrin with few collagen fibrils (Fig. 7A). Knowing that the cells are responsive to exogenous TGFβ3, we added TGFβ3 (0.02 μg/ml) to the BM-MNC-fibrin constructs and continued culture for an additional 7 days. The results showed that the addition of exogenous TGFβ3 had resulted in the synthesis of an extracellular matrix containing narrow-diameter collagen fibrils and the cells exhibited fibripositors (Fig. 7B).

## 3 Discussion

In this study we showed that culturing MSCs in fixed-length 3D fibrin gels for 7 days is sufficient to direct MSCs towards an embryonic tendon-like phenotype. The cells deposited parallel arrays of narrow-diameter collagen fibrils, some of which occurred in fibripositors. We showed that MSCs contracted a 3D fibrin gel, upregulated the expression of TGFβ3 at the time of full contraction, and was responsive to self-generated TGFβ signaling. The addition of a TGFβ signaling inhibitor stopped the phosphorylation of Smad2 and prevented the synthesis of collagen fibrils. We also showed that the addition of exogenous TGFβ3 to BM-MNCs is sufficient to induce the cells to synthesize a tendon-like matrix.

The MSCs used in this study were positive for CD44 (the hyaluronan receptor), CD90 (Thy1) and CD105 (endoglin) and negative for CD11b. CD11b was a useful negative marker because it is expressed on haematopoietic cells and not on MSCs (Colter et al., 2001). In further experiments we showed that MSCs cultured in monolayer could be induced to form adipogenic, chondrogenic and osteogenic cells, as expected. The presence of FABP-4, an adipose-specific cytosolic chaperone, and lipid vacuoles was used to confirm adipogenic differentiation (Lapsys et al., 2000). Osteogenic differentiation by MSCs was confirmed by the staining for osteocalcin, a non-collagenous ECM protein, and for calcium deposition, both of which are markers of osteoblasts (Owen et al., 1990). We noted that uninduced MSCs also expressed low levels of osteocalcin because they maintain their bone marrow-derived phenotype through *in vitro* expansion (Yamagiwa et al., 2001). In addition to expression of osteoblast markers, MSCs cultured in osteogenic media detached from the coverslip, which is another feature of osteogenic differentiation (Kitano et al., 2000).

To examine the effects of cell-induced uniaxial tension on the differentiation state of the MSCs, we cultured the cells in fixed-length fibrin gels. Noteworthy, embryonic chick tendon fibroblasts maintain their fibripositor phenotype when cultured in fixed-length 3D fibrin gels but not when cultured in monolayer on tissue culture plastic (Kapacee et al., 2008). The results showed that the MSCs synthesized narrow-diameter collagen fibrils and exhibited fibripositors, which are both hallmark features of embryonic tendon cells (for examples see (Birk and Trelstad, 1986; Canty et al., 2004)).

We showed that culturing the cells in the fixed-length 3D tendon-like constructs dampened the ability of the cells to undergo osteogenic induction. It was recently shown that substrate stiffness can affect hMSC commitment to tissue-specific lineages (Engler et al., 2006). However, it was not known if the pre-committed MSCs retained their multipotency. We noticed that some of the cells maintained the potential for adipogenic differentiation after being cultured in 3D tendon-like constructs. It has been reported that fibroblastic cells, such as tenocytes, have the potential for adipogenic differentiation (Mackley et al., 2006; Wolf et al., 2003). Conversely, some investigators have raised the question that sub-populations of fibroblasts are in fact stem cells; stem cells isolated from a niche within the tendon possess stem cell surface markers and multipotency (Bi et al., 2007; de Mos et al., 2009) (Salingcarnboriboon et al., 2003). If multipotent MSCs were still present, even if pre-committed to an embryonic tendon lineage, they might be expected to undergo osteogenic differentiation (Bi et al., 2007; de Mos et al., 2009) (c). However, the lack of alizarin red S staining indicated that tension abrogated the osteogenic potential of MSCs isolated from the constructs, which strongly suggests that naïve MSCs were no longer present after 7 days of culture under tension. Taken together, the differentiation results showed that the ability to induce MSC differentiation *in vitro* was dampened by continued culture under tension, which raises the intriguing possibility that uniaxial tension during limb development may contribute to the progression of stem cells along a tendon lineage.

Quantitative PCR showed that the levels of col1a1 gene expression increased ~ 5-fold when cells were transferred from monolayer to 3D constructs, which was consistent with the assembly of a collagen fibril matrix. Further analysis showed that TGFβ3, integrins β1, β5 and β8, as well as Smad2 were also upregulated. We included scleraxis in our analyses. Scleraxis is a member of the basic helix–loop–helix superfamily of transcription factors, it is a marker for progenitor cells of sclerotomal origin, and its expression has been localised in progenitors of connective tissue lineage such as tendon and ligament (Schweitzer et al., 2001). The levels of expression of scleraxis mRNA were statistically unchanged when MSCs were transferred from monolayer to 3D gels. Other studies have located scleraxis to chondro-progenitor condensations (Cserjesi et al., 1995; Hargus et al., 2008) and in osteoblasts (Liu et al., 1996), which has been used to suggest that scleraxis can have additional functions not restricted to tendon development. For example, studies have shown that over expression of scleraxis leads to an increase in aggrecan deposition and synthesis, as well as collagen II and osteopontin mRNA levels, which are cartilage- or bone-specific proteins (Liu et al., 1997). Our results suggest that low levels of scleraxis are most probably sufficient to direct stem cells along a tendon differentiation path.

An important lead from the Q-PCR experiments was the upregulation of TGFβ3 gene expression at the time of maximal contraction of the constructs. This observation led to a series of experiments that showed the phosphorylation of Smad2 in MSCs in tendon-like constructs at the time of maximal contraction. In further experiments we showed that inhibition of TGFβ binding to its receptor (by the inhibitor SB-431542) blocked the phosphorylation of Smad2 and blocked the formation of collagen fibrils. We showed that BM-MNCs were not effective in forming a collagen fibril-containing tendon-like construct. After 14 days in culture, the cells had contracted the fibrin to a thin strand that did not contain collagen fibrils. However, the addition of exogenous TGFβ3 resulted in the phosphorylation of Smad2, the synthesis of collagen fibrils, and the subsequent formation of a tendon-like construct. Three conclusions were drawn from these results. First, self-generated Smad-dependent signaling was sufficient for MSC tendon induction. Second, TGFβ signaling only occurred when the constructs were fully contracted. Third, exogenous TGFβ3 was sufficient to induce BM-MNCs to synthesize a tendon matrix. In a previous study we showed that embryonic chick tendon cells maintained their embryonic phenotype only when cultured under conditions of linear tension (Kapacee et al., 2008); loss of tension resulted in disorganization of the collagen fibrils and loss of cell fibripositors. Therefore, it is likely that embryonic tendon induction and maintenance requires a delicate balance between uniaxial tension and TGFβ3 signaling.

TGFβ signaling requires that the mature TGFβ molecule be released from sequestration in the ECM. The mechanism of TGFβ3 release in the tendon-like constructs is not known, and could involve a variety of mechanisms involving proteolytic and non-proteolytic events. Interestingly, rapid synthesis of collagen fibrils and phosphorylation of Smad2 coincided with the time of maximum contraction of the constructs. Hinz and co-workers have shown that contraction of myofibroblasts functions as a mechanism to directly activate TGFβ1 from self-generated stores in the ECM (Wipff et al., 2007). These workers showed that the process was inhibited either by antagonizing integrins or reducing ECM compliance and was independent of protease activity. It is possible, therefore, that the same mechanism occurs in tendon-like constructs in that contraction of the matrix by the MSCs activates stores of self-generated TGFβ3. Interestingly, our Q-PCR data showed no significant changes in levels of expression of biglycan or decorin (both of which bind TGFβ1 with greater avidity than TGFβ3) or of TGFβ1, LTBP-1 or thrombospondin-1. Significant increases in expression occurred for the α1(I) chain of type I collagen and integrins β1, β5 and β8 at the time of maximal contraction, collagen fibril assembly and reorganization of the cytoskeleton at the sites of fibripositor formation at the surface of the cells. Aluwihare and co-workers showed that mice lacking activity of αvβ6 and αvβ8 reproduce the abnormalities of *tgfb1*- and *tgfb3*-null mice (Aluwihare et al., 2009). Thus, it seems likely that the effects observed in our study are a combination of cell-force induced release of TGFβ3 stores from the matrix combined with integrin-mediated dissociation of the LAP-TGFβ3 complex. Whether or not one or more of these integrins is required for the release of TGFβ3 from the ECM (*in vitro* and *in vivo*) and if there is cross-talk with pathways involved in collagen fibril assembly and actin reorganization at sites of fibripositor formation, is a focus of further work that will build upon the results shown here.

## 4 Materials and methods

### 4.1 Source of materials

All chemicals were obtained from Sigma-Aldrich, UK; sterilized culture reagents and cell culture materials were obtained from Lonza BioWhittaker, UK and BD Falcon, UK, respectively, unless otherwise stated.

### 4.2 Isolation of human mesenchymal stem cells (MSCs)

Characterized human bone marrow stem cells (from two donors 20 and 22 years of age) were obtained from Lonza Biosciences (Berkshire, UK, http://www.lonza.com) and cultured in Mesencult medium supplemented with 10% fetal calf serum and 1 ng/ml basic fibroblast growth factor (R&D Systems Europe Ltd., UK). Cultures were maintained in a humid atmosphere of 5% CO_2_/95% air at 37 °C. Once cells had reached confluence they were passaged using trypsin/EDTA at a split ratio of 1:3. Experiments were performed using cells at P3, and all experiments were repeated with cells from all four donors.

### 4.3 Isolation of human bone marrow-derived mononuclear cells (BM-MNCs)

Cells from two donors aged 20 and 44 years were obtained from Lonza and selected by adherence over 24 h to tissue culture plastic. The cells were expanded in monolayer culture in Mesenchymal Stem Cell Medium (Lonza) supplemented with 5 ng/ml fibroblast growth factor-2 (R&D Systems, Ltd., Abingdon, UK, http://www.rndsystems.com) as previously described(Murdoch et al., 2007). Twenty-four hours after plating, the non-adherent cells were removed and replaced with Dulbecco's modified Eagle's medium containing 4500 mg/L d-Glucose, non-essential amino acids and 110 mg/L sodium pyruvate (DMEM4), supplemented with 100 U/ml penicillin, 100 μg/ml streptomycin, 2 mM l-glutamine and 10% fetal calf serum (FCS). Colonies formed after plating were designated as passage 0 (P0).

### 4.4 Lineage analysis

Cells at passage 2–3 were seeded on glass coverslips in a 24-well plate. For adipogenic induction, cells were seeded at densities of 3.7 × 10^4^ cells per well. The cells were incubated in alpha-minimum essential medium (α-MEM) supplemented with 100 U/ml penicillin, 100 μg/ml streptomycin, 2 mM l-glutamine, 10% FCS and adipogenic supplement (hydrocortisone, isobutyl-methylxanthine and indomethacin; R&D Systems Europe Ltd., UK) at 37 °C in 5% CO_2_. For osteogenic induction, cells were seeded at densities of 7.4 × 10^4^ cells per well. The cells were incubated in α-MEM supplemented with 100 U/ml penicillin, 100 μg/ml streptomycin, 2 mM l-glutamine, 10% FCS and osteogenic supplement (dexamethasone, ascorbate-phosphate and β-glycerolphosphate; R&D Systems Europe Ltd., UK) at 37 °C in 5% CO_2_. For both lineages, the cells were cultured for 21 days, with media changes every 4 days.

### 4.5 Adipogenic differentiation

Adipogenic differentiation was assessed using Oil Red O staining for lipids and immunocytochemistry for fatty acid binding protein-4 (FABP-4). Cells were fixed in 2% paraformaldehyde (PFA), permeabilised 0.5% Triton X-100 in PBS, washed with PBS and stained with Oil Red O for 10 min. Cells were counter-stained with haemotoxylin and mounted using non-xylene based Vector Shield with DAPI (Vector Laboratories, UK). All light microscopy images were taken using Zeiss Axioplan 2 microscope (Zeiss, Germany). For immunocytochemistry of FABP-4, cells were fixed in 4% PFA, then blocked and permeabilised with 0.3% Triton X-100, 1% BSA and 10% normal donkey serum (Jackson Immuno Research, USA) in PBS for 45 min at room temperature. Cells were incubated with 10 μg/ml primary antibody, goat anti-mouse FABP-4, overnight at 4 °C (R&D Systems Europe Ltd.). The cells were then washed extensively with 1% BSA in PBS before incubation with 1 μg/ml secondary antibody, donkey anti-goat conjugated with FITC (Santa Cruz Biotechnology, USA) for 1 h at room temperature in the dark. Cells were mounted onto glass slides using Vector Shield with DAPI. All fluorescent microscopy images were taken using Olympus BX51 (Olympus, USA).

### 4.6 Osteogenic differentiation

Osteogenic differentiation of MSCs was assessed using Alizarin Red stain for calcium deposits and immunocytochemistry for osteocalcin. Cells were fixed with 2% PFA, washed with distilled water and stained with Alizarin Red for 20 min at room temperature. Cells were mounted onto glass slides using Pertex (Cellpath Ltd., UK). Images were collected as above. For immunocytochemistry of osteocalcin, a similar protocol was used as for the localisation FABP-4 as above. Cells incubated with 10 μg/ml primary antibody, mouse anti-human osteocalcin (R&D Systems Europe Ltd.). The cells were washed extensively with 1% BSA in PBS and incubated with 5 μg/ml secondary antibody, goat anti-mouse conjugated with Alexa 488 (Molecular Probes, CA, USA) for 1 h at room temperature in the dark. Images were collected as above.

### 4.7 Chondrogenic differentiation

Chondrogenic differentiation was performed as described previously (Murdoch et al., 2007). In brief, isolated stem cells were resuspended in chondrogenic culture medium consisting of high glucose Dulbecco's modified Eagle's medium supplemented with 100 μg/ml sodium pyruvate (Lonza), 10 ng/ml TGFβ3 (R&D Systems), 100 nM dexamethasone, 1× ITS + 1 premix, 40 μg/ml proline, and 25 μg/ml ascorbate-2-phosphate (all from Sigma-Aldrich, Poole, U.K., http://www.sigmaaldrich.com). Aliquots of cells (5 × 10^5^) were spun in 15-ml polypropylene tubes (1 ml of medium, 240 ×*g*, 5 min). Culture medium was replaced every second day for up to 28 days.

### 4.8 Flow cytometry

All steps were performed on a rotating platform at 4 °C. Approximately 1 × 10^6^ cells per treatment were blocked with 1% BSA/PBS for 1 h and incubated with primary antibody in PBS for 1 h. Unbound antibody was cleared in three 5 minute PBS washes. The cell suspensions were then incubated with secondary antibody diluted in PBS for 1 h in the dark. Analyses of cell surface epitopes were performed using a Dako CyAn™ ADP flow cytometry analyzer (Becton Dickinson (Coulter), CA, USA). The samples were kept on ice to maintain integrity and minimize cell death. Dead cells were excluded by labeling with 7-aminoactinomycin, whilst viable cells were gated by their forward and side scatter characteristics. Primary antibodies were used at a dilution of 1:100: mouse anti-human CD44 (Dako Cytomation, Cambridge, UK), mouse anti-human CD90 (BD Pharmingen, CA, USA) and mouse anti-human CD105 (Abcam, Cambridge, UK), rat anti-human CD11b (BD Pharmingen, CA, USA). Secondary antibodies were used at a dilution of 1:1000: goat anti-mouse IgG conjugated with FITC and goat anti-rat IgG conjugated with FITC (Serotec, Oxfordshire, UK).

### 4.9 Preparation of culture plates

Samples were prepared as described previously (Kapacee et al., 2008). In brief, wells of a six well plate were coated with Sylgard (type 184 silicone elastomer, Dow Chemical, Midland, MI, USA) and incubated overnight at 55 °C to induce polymerisation. Fixed-position posts were created in each well by pinning minutiens insect pins (0.1 mm diameter, Fine Science Tools GmbH, Germany) onto size 0 silk suture (metric size, 3.5; Ethican, Somerville, NJ, USA) positioned 10 mm apart. The plates were sterilized by immersing in 100% ethanol and exposure to ultraviolet irradiation in a biological safety cabinet for 60 min. Cells were seeded using 7.5 × 10^5^ cells per well, suspended in 20 mg/ml fibrinogen and 200 U/ml thrombin (Sigma, St Louis, MO, USA).

### 4.10 Isolation of cells from fibrin constructs

Human MSCs cultured in the 3D fibrin constructs were released with trypsin (37,000 U) and bacterial collagenase (522 U) in DMEM at 37 °C for 2 h. Cells were passed through a 70 μm cell strainer (BD Biosciences, UK), collected by centrifugation (240 ×*g* for 5 min) and washed three times in PBS. Cells were resuspended in DMEM4 with 100 U/ml penicillin, 100 μg/ml streptomycin, 2 mM l-glutamine and 10% FCS and tested for their multipotency or resuspended in 1% BSA/PBS for surface epitope analysis.

### 4.11 RNA isolation and cDNA synthesis

RNA was isolated from hMSC cultured on monolayer and in the fibrin constructs using the TRIzol reagent™ (Invitrogen, CA, USA). Briefly, for monolayer cultures, 1 ml of TRIzol reagent was added to 6 × 10^6^ cells and the cells lysed by repetitive pipetting. Constructs were homogenized in TRIzol reagent and transferred to a dismembrator chamber, which was then rapidly equilibrated to − 196 °C by immersion in liquid nitrogen. Once frozen, samples were dismembrated in the Mikro-dismembrator-S (Sartorius Stedim Biotech) at 2000 rpm for 90 s, performed two times, and then the mixture was carefully transferred to a 1.5 ml Eppendorf tube. RNAs were extracted from the samples following the manufacturer's instructions (Invitrogen, CA, USA). Isolated RNA was DNAse treated and cDNA was transcribed from 2 μg of RNA with TaqMan reverse transcriptase (RT) polymerase (Applied Biosystems, UK) using an oligo(dT)_16_ primer.

### 4.12 Real-time PCR

cDNA prepared from monolayer cultures of MSCs and used as template to validate the following human primer sequences: GAPDH: forward primer ATGGGGAAGGTGAAGGTCG, reverse primer, TAAAAGCAGCCCTGGTGACC; colIa1: forward primer CAGCCGCTTCACCTACAGC; reverse primer TTTTGTATTCAATCACTGTCTTGCC; scleraxis: forward primer CCTGAACATCTGGGAAATTTAATTTTAC, reverse primer CGCCAAGGCACCTCCTT(Schulze-Tanzil et al., 2004); tenascin C: forward primer TTTCTGACATAACTCCCGAGAGC, reverse primer AGATATGGGCAGTTCGTTCAGC(Ogawa et al., 2005); fibronectin: forward primer ACCAACCTACGGATGACTCG, reverse primer, GCTCATCATCTGGCCATTTT. decorin: forward primer AATTGAAAATGGGGCTTTCC, reverse primer GCCATTGTCAACAGCAGAGA; latent TGFβ binding protein (LTBP)-1: forward primer TCCTGGGGCTTTAACAAATG, reverse primer CGATAGCTGCCCATGGTATT; TGFβ1: forward primer CACGTGGAGCTGTACCAGAA, reverse primer GAACCCGTTGATGTCCACTT; TGFβ3: forward primer GGAATGAGCAGAGGATCGAG, reverse primer ATTGGGCTGAAAGGTGTGAC; fibromodulin: forward primer CCACTTCACCCACTCCACTT, reverse primer CTGGTGACCTCCAATCTGGT; biglycan: forward primer GGACTCTGTCACACCCACCT, reverse primer AGCTCGGAGATGTCGTTGTT; thrombospondin-1: forward primer TTGTCTTTGGAACCACACCA, reverse primer CTGGACAGCTCATCACAGGA; fibrillin-1: forward primer AGCCTGGGGTACTGAGGAAT, reverse primer TGCACTTAAAGCTGCCAATG; Smad2: forward primer CGAAATGCCACGGTAGAAAT, reverse primer CCAGAAGAGCAGCAAATTCC; integrin αv: forward primer ACTGGGAGCACAAGGAGAACC, reverse primer CCGCTTAGTGATGAGATGGTC; integrin β1: forward primer CATCTGCGAGTGTGGTGTCT, reverse primer GGGGTAATTTGTCCCGACTT; integrin β5: forward primer ACAAGGGAGTCCTCTGCTCA, reverse primer GGGGCACTTCTCACACATCT; integrin β6: forward primer TGCGACCATCAGTGAAGAAG, reverse primer GACAACCCCGATGAGAAGAA; integrin β8: forward primer ACCCCTCACTAGGCCAACTT, reverse primer GCCTTGTACCTGGTTTTCCA; and bmp1/tolloid: forward primer CTGTGAGTGGGTCATTGTGG, reverse primer GGTGTCATCCGAGTGGAACT. Primers were obtained from MWG Biotech AG (Ebersberg, Germany). Amplified products were separated by 1.5% agarose gel electrophoresis and visualized by ethidium bromide staining. Reaction products were gel purified and analysed by DNA sequencing to confirm their identity.

For real-time PCR, cDNA prepared from monolayer and 3D fibrin cultures of MSCs was amplified by PCR in 12 μl reaction volumes on an Abi prism 7000 detection system (Applied Biosystems, UK) using a SYBR Green Core kit (Applied Biosystems, UK). Relative expression levels were normalised using glyceraldehyde 3-phosphate dehydrogenase (GAPDH) and calculated using the 2^− ΔΔCt^ method (Livak and Schmittgen, 2001).

### 4.13 Western blotting

Adherent cells on monolayer or cells in the fibrin gels were lysed using 5× RIPA buffer (750 mM NaCl, 250 mM Tris pH 7.4, 25 mM EDTA, 5% NP40, 5% DOC, 0.5% SDS, with freshly added protease and phosphatase inhibitors). Whole cells lysates were separated by discontinuous SDS-PAGE and electroblotted onto polyvinylidene diflouride membrane. Primary antibodies used include, Smad2 (mouse mAb, 1:500, Cell Signaling, MA, USA), P-Smad2 (rabbit mAb, 1:500, Cell Signaling, MA, USA) and GAPDH (mAb, 1:10,000, Sigma). Secondary antibodies used include, peroxidase conjugated donkey anti-mouse IgG and peroxidase conjugated donkey anti-rabbit IgG (1:1000, Stratech, Suffolk, England) Chemiluminescence was detected with SuperSignal West Dura Extended Duration Substrate (Pierce Biotechnology, Inc.). Fuji medical X-ray film (Fujifilm) was used to detect the light emitted from bound HRP and the films were developed using a film processor (IGP Compact_2_; Silvermatic) according to the manufacturer's instructions.

### 4.14 Electron microscopy

Samples were prepared for electron microscopy as described previously (Canty et al., 2004). Sections were examined with an FEI Tecnai 12 Biotwin transmission EM. Images were recorded on 4498 film (Kodak) and subsequently digitized using an Imacon Flextight 848 scanner (Precision Camera and Video).

## Source of funding

The work was supported by Wellcome Trust grant 076939/Z/05/Z (to K.E.K.). C-Y.C.Y. is a recipient of a Biotechnology, Biological Sciences Research Council (BBSRC, UK) PhD studentship.

Supplementary materials related to this article can be found online at doi:10.1016/j.matbio.2010.08.005.

The following are the supplementary materials related to this article.

Supplemental 1 Analysis of hMSC surface epitopes using flow cytometry. A, total cell population of unlabeled cells display forward scatter (FS) and slide scatter (SS). B, selective gating of live cells. C, analysis for CD90, CD44, CD105, CD34 and CD11b markers on MSCs cultured in monolayer, using secondary antibody only (control IgG) or with both primary (1°) and secondary (2°) antibodies.

Supplemental 2 Human MSCs respond to adipogenic and osteogenic induction. A–L, differentiation studies with cells prior to culture in tendon-like constructs. M–P, differentiation studies with cells released from tendon-like constructs 7 days post-contraction. A, B; cells cultured for 21 days in control and adipogenic differentiation medium and examined by cytohistochemistry using Oil Red O to detect lipid droplets. C, D; cells cultured for 21 days in control and adipogenic differentiation medium and examined by immunocytochemistry using a primary antibody that recognizes fatty acid binding protein (FABP)-4. E, F; cells cultured for 21 days in control and osteogenic differentiation medium and stained with alizarin red S to detect calcified deposits. G, H; cells cultured for 21 days in control and osteogenic differentiation medium and examined by immunocytochemistry using a primary antibody that recognizes osteocalcin. I, J; cells cultured for 28 days in pellet cultures in control and chondrogenic differentiation medium and examined by haemotoxylin and eosin (H&E) staining. K, L; cells cultured for 28 days in pellet cultures in control and chondrogenic differentiation medium and examined by safranin O staining. M, N; cells recovered from tendon-like constructs and cultured for 21 days in control and osteogenic differentiation medium and stained with alizarin red S to detect calcified deposits. O, P; cells recovered from tendon-like constructs and cultured for 21 days in control and osteogenic differentiation medium and examined by immunocytochemistry using a primary antibody that recognizes osteocalcin. Bar = 10 μm.

Supplemental 3 Ultrastructural analysis of MSCs cultured on fibrin gels. A, cells treated with thrombin under identical conditions as those used to prepare tendon-like constructs but the cells were cultured ‘on’ pre-formed fibrin for 7 days. B, cells cultured on pre-formed fibrin for 7 days. Note the absence of collagen fibrils (compare these data with those shown in Fig. 7).

Supplemental 4 Quantitative PCR comparison of gene expression in MSCs cultured in monolayer and in tendon-like constructs, relative to expression of the gene for GAPDH. No significant changes were observed for the genes shown.
