# Supplementary figures and images for: Synthesis of embryonic tendon-like tissue by human marrow stromal/mesenchymal stem cells requires a three-dimensional environment and transforming growth factor β3
Source: Matrix Biol. 2010 Oct;29(8):668–77. doi: 10.1016/j.matbio.2010.08.005 (PMC3611595; doi:10.1016/j.matbio.2010.08.005)

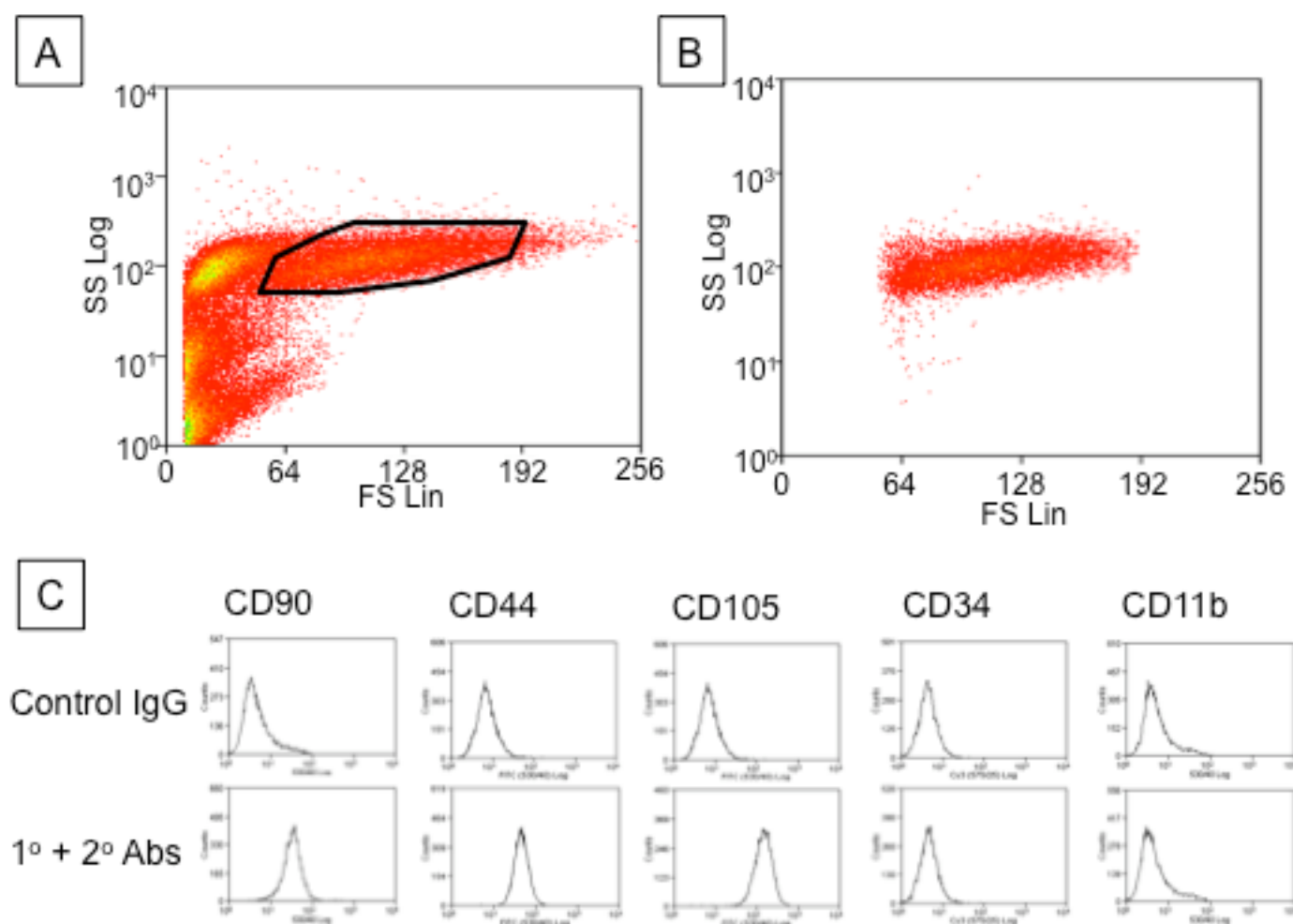

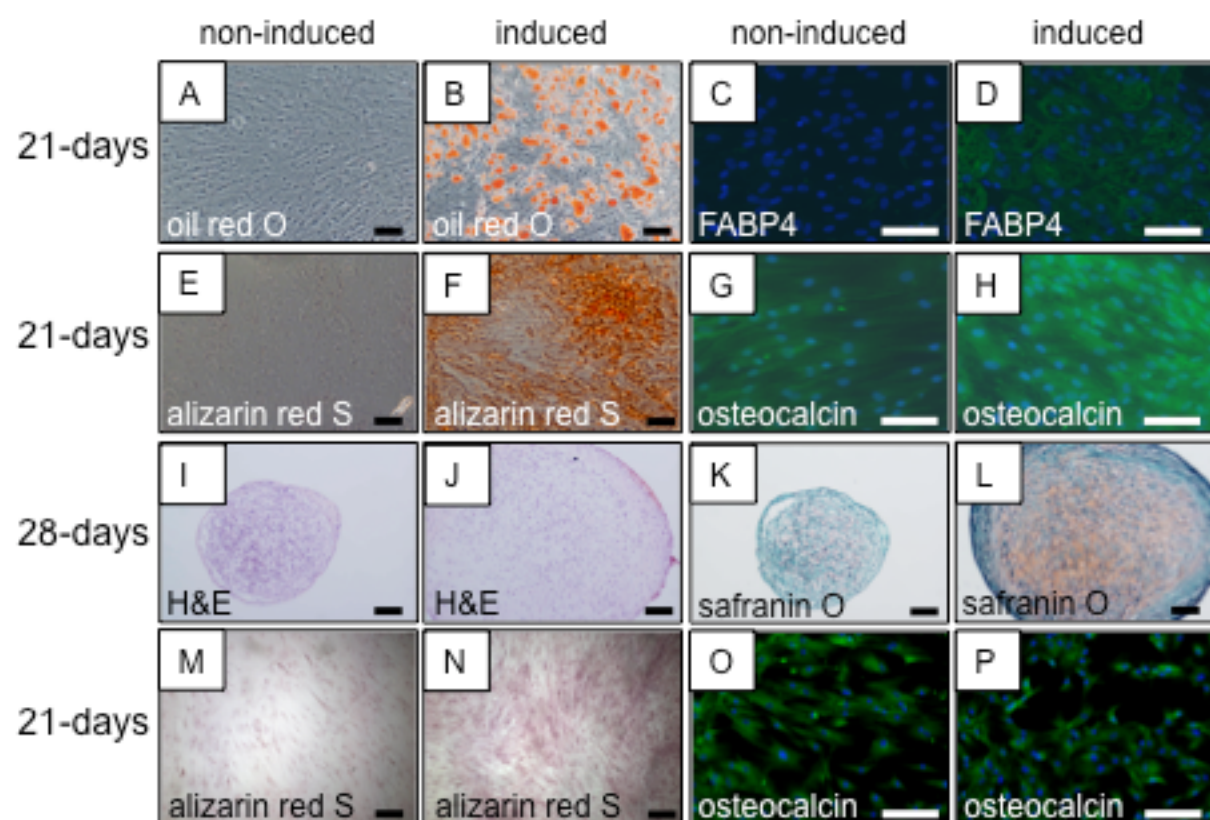

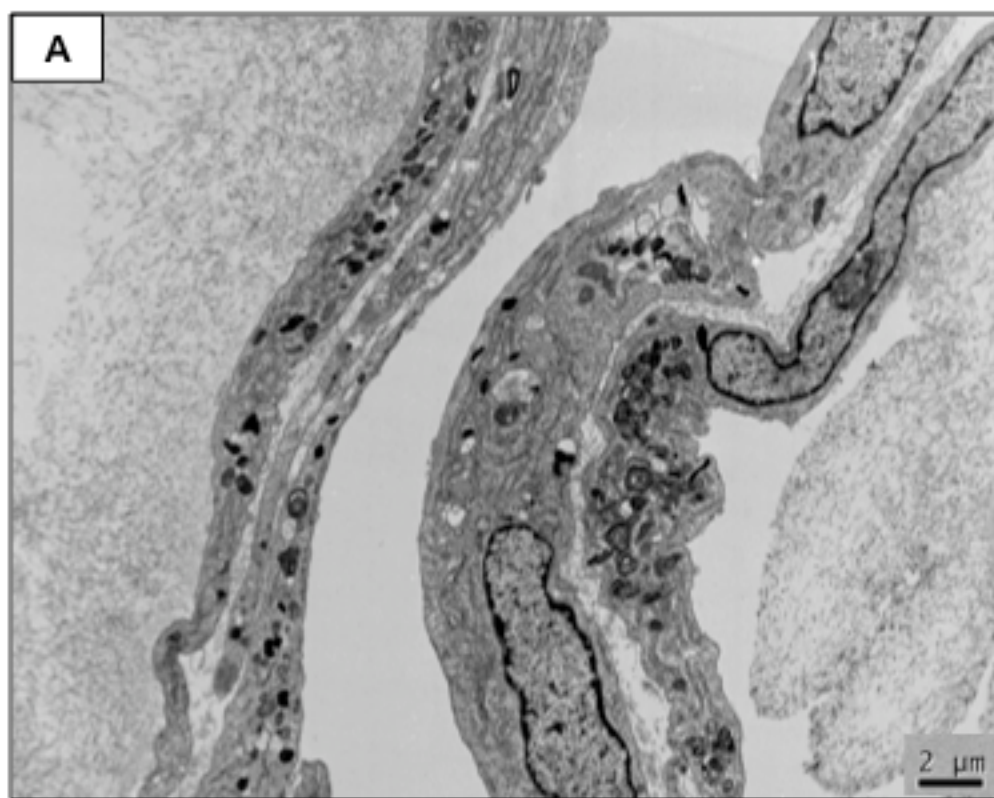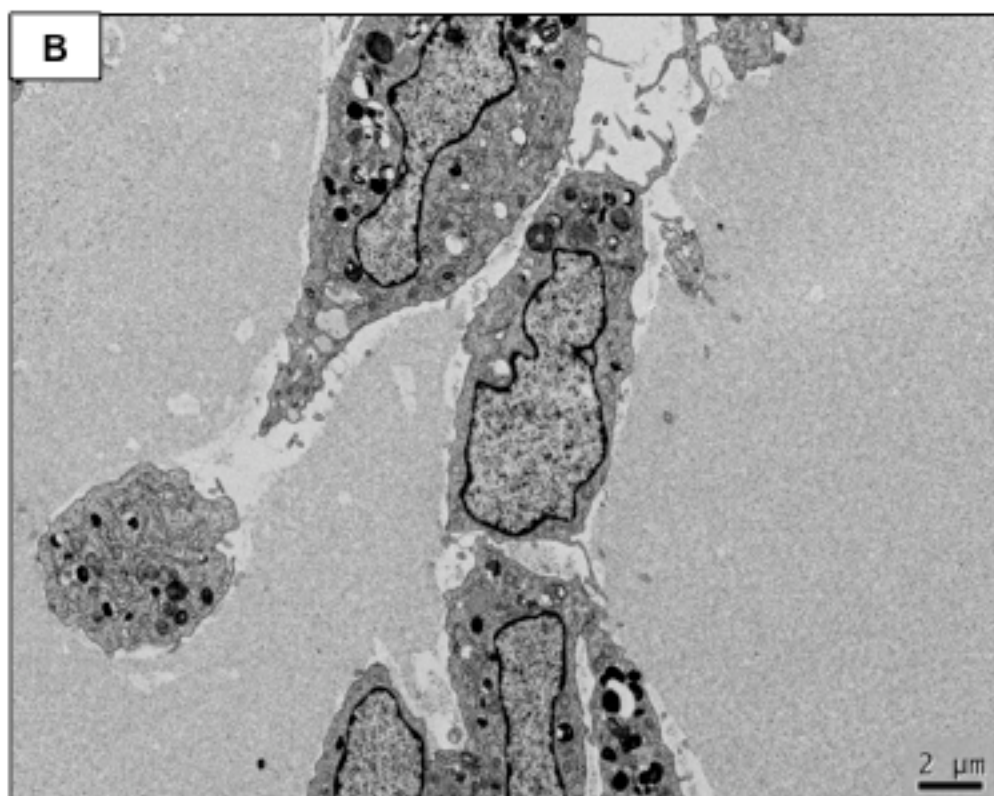

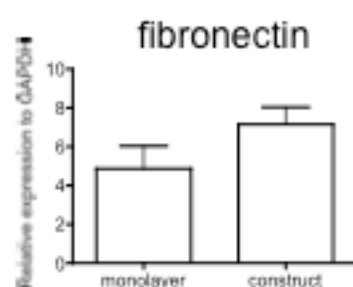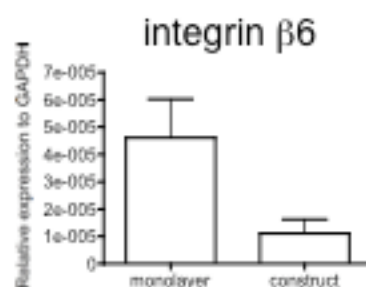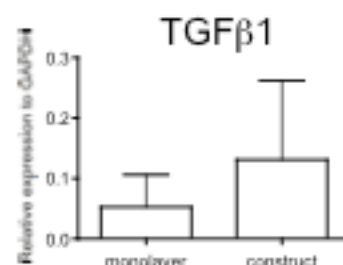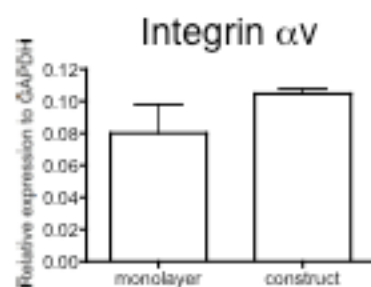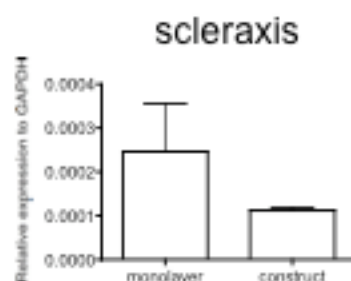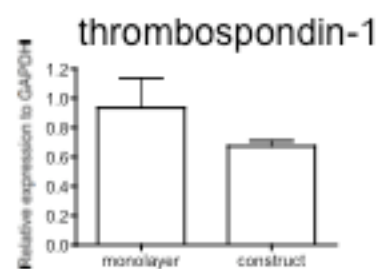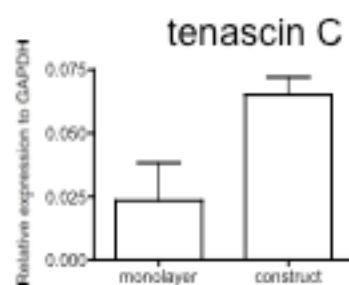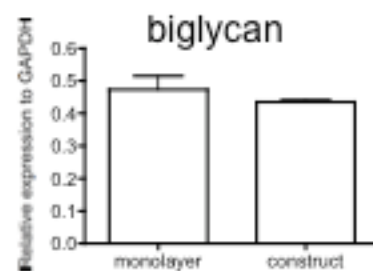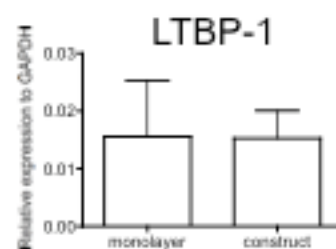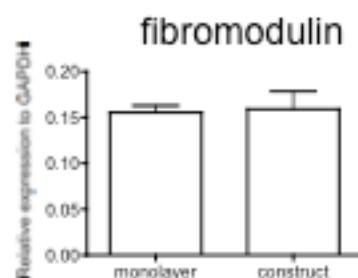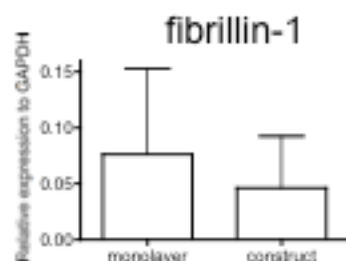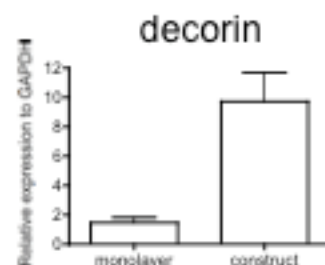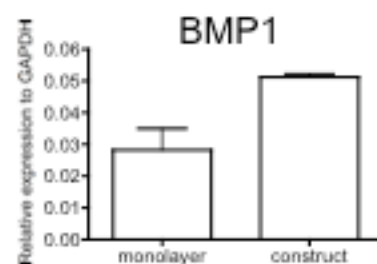

Supplement: Supplemental 1 — Analysis of hMSC surface epitopes using flow cytometry. A, total cell population of unlabeled cells display forward scatter (FS) and slide scatter (SS). B, selective gating of live cells. C, analysis for CD90, CD44, CD105, CD34 and CD11b markers on MSCs cultured in monolayer, using secondary antibody only (control IgG) or with both primary (1°) and secondary (2°) antibodies. [file mmc1.pdf]
